# Evaluation of Gridded Precipitation Data for Driving SWAT Model in Area Upstream of Three Gorges Reservoir

**DOI:** 10.1371/journal.pone.0112725

**Published:** 2014-11-19

**Authors:** Yan Yang, Guoqiang Wang, Lijing Wang, Jingshan Yu, Zongxue Xu

**Affiliations:** 1 College of Water Sciences, Beijing Normal University, Beijing 100875, China; 2 United Graduate School of Agricultural Science, Gifu University, 1-1 Yanagido, Gifu, 501-1193, Japan; 3 State Environmental Protection Key Laboratory of Drinking Water Source Protection, Chinese Research Academy of Environmental Sciences, Beijing 100012, China; Tennessee State University, United States of America

## Abstract

Gridded precipitation data are becoming an important source for driving hydrologic models to achieve stable and valid simulation results in different regions. Thus, evaluating different sources of precipitation data is important for improving the applicability of gridded data. In this study, we used three gridded rainfall datasets: 1) National Centers for Environmental Prediction - Climate Forecast System Reanalysis (NCEP-CFSR); 2) Asian Precipitation - Highly-Resolved Observational Data Integration Towards Evaluation (APHRODITE); and 3) China trend - surface reanalysis (trend surface) data. These are compared with monitoring precipitation data for driving the Soil and Water Assessment Tool in two basins upstream of Three Gorges Reservoir (TGR) in China. The results of one test basin with significant topographic influence indicates that all the gridded data have poor abilities in reproducing hydrologic processes with the topographic influence on precipitation quantity and distribution. However, in a relatively flat test basin, the APHRODITE and trend surface data can give stable and desirable results. The results of this study suggest that precipitation data for future applications should be considered comprehensively in the TGR area, including the influence of data density and topography.

## Introduction

Precipitation data are generally recognized as the most important driving data for hydrologic models. However, constrained by the sparse distribution of observation stations, the applicability of such models is limited. With development of modern observation and massive computing technologies, the estimation of precipitation based on combination of multi-source data (historical observed, radar and satellite) has become a feasible means for extending model applications. Various data are published for global or regional modeling, including climate change, global water cycle, and ecology modeling [1]–[3]. For example, Chappell [4] used TRMM data, station observed data and a kernel-based statistical blending algorithm to produce 5-km resolution gridded precipitation data for Australia. Additionally, Li et al. [5] and Huang et al. [6] used spline interpolation and the trend surface methods to generate 5-km resolution gridded precipitation data for New Zealand and China. With the expanded application requirements, these data have been widely used for hydrologic modeling in various studies and regions [7], [8], [9].

However, errors in the gridded precipitation data have a high probability for inducing errors and uncertainties in hydrologic simulations. Thus, validations and evaluations of these gridded data require attention. The gridded data are generally compared with observed data. For example, Wang and Zeng [10] evaluated six reanalysis climate data products (MERRA, NCEP/NCAR-1, NCEP-CFSR, ERA-40, ERA-Interim, and GLDAS) versus in situ measurements at 63 weather stations on the Tibetan Plateau, showing that the NCEP-CFSR (National Centers for Environmental Prediction Climate Forecast System Reanalysis) data had the best overall performance. Bao et al. [11] successfully evaluated four different data (NCEP-NCAR reanalysis, NCEP-CFSR, ERA-40, and ERA-Interim) products based on an enhanced observed network, and the results showed that the performance of NCEP-CFSR and ERA-Interim data were superior for the Tibetan Plateau. Areal accuracy and validity of gridded precipitation data are evaluated using a hydrologic model. For example, Fuka et al. [12] presented a method using the NCEP-CFSR global meteorological data to model five watersheds representing different hydro-climate regimes. The results proved that NCEP-CFSR precipitation can provide runoff simulations that are as good as or better than traditional weather gauging stations. Vu et al. [13] compared five different sources of rainfall data for the Vietem River basin using the Soil and Water Assessment Tool (SWAT) model, finding that all the gridded data could capture hydrologic processes in this tropical basin well. For model driving, most gridded precipitation data are developed for application to much larger basins, for example continental or global scale [1], [3], and their applicability is always validated in such large basins [12], [13], [14]. In small basins, it is commonly recognized that precipitation is mainly influenced by topographic, wind direction, hill aspect, and other factors, and the creation or reanalysis of precipitation data in such small basin usually required more detailed information compared with the data in large scale basins [15], [16], [17]. However, the applicability of various gridded data to such basins requires further investigation.

The Three Gorges Reservoir (TGR) in the middle reach of the Yangtze River is the largest hydropower project in the world. Although TGR has the benefit of flood control and electric generation, its influence on hydrologic processes, its alteration of ecological systems and the environment has attracted public attention. Hydrologic models have been applied to this region to analyze these influences at different spatiotemporal scales [18]–[21]. However, limited by sparse observation stations in the mountains and missing observations in immigration regions, the applicability of those models remains limited in this study area. Additionally, water resource analysis and pollution control in the TGR area are needed for local tributary basins, which have strong topographic variation, relatively small scales, and a lack of dense observed data. Although global or regional gridded precipitation data are probable sources for model simulation in such regions, the applicability of these data require further study.

Thus, the main objective of this study was to evaluate various gridded precipitation data by both station validation and model driving tests for the area upstream of TGR. In this area, two subbasins of the Pengxi River were selected as the test basin. NCEP-CFSR, Asian Precipitation - Highly-Resolved Observational Data Integration Towards Evaluation (APHRODITE), and trend surface data were used for the evaluation. These gridded data were compared with observed data. Then, simulation results of the data were appraised based on quantitative evaluation indices of model simulation and uncertainties. Spatial density and receptiveness of the topography for the various data are also discussed.

## Study Area and Data

### Study basins

**Figure 1:**
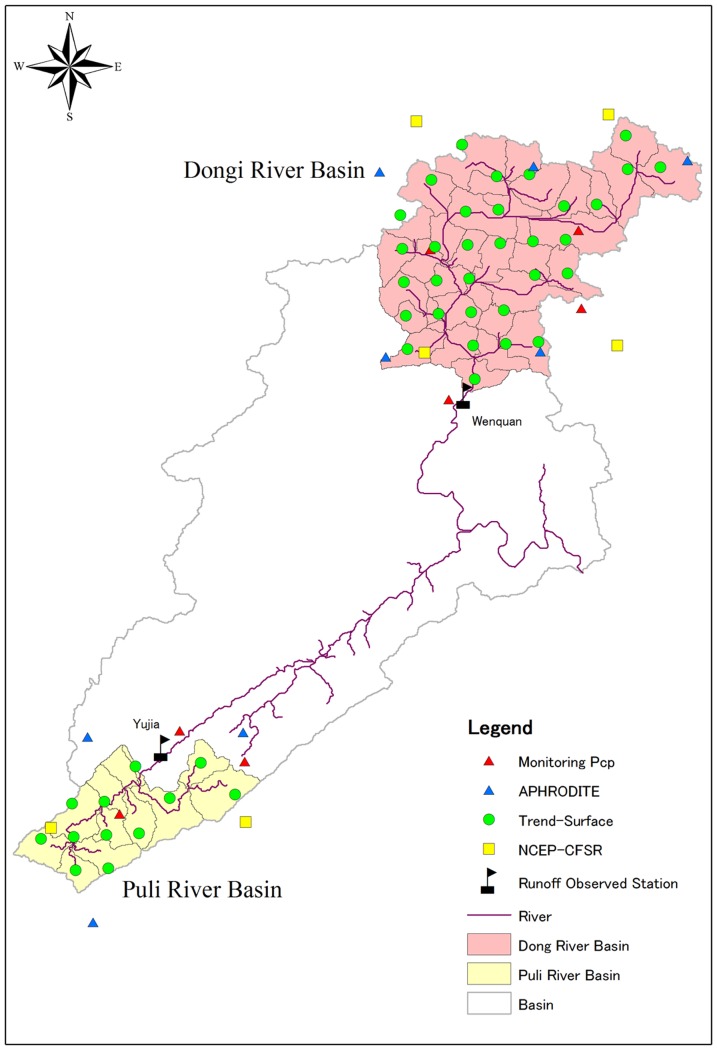
Basic geo-information of the test basins.

We selected two test basins (of the Dong and Puli rivers) for study. These basins belong to the basin of the Pengxi River, one of the main tributaries on the north shore of TGR [21]. In this region, average annual precipitation is 1100–1500 mm. Daily discharge data were collected from gauging stations in the two basins (Figure 1; Table 1), Wenquan in the Dong River basin and Yujia in the Puli River basin. Wenquan is at the outlet of the Dong River basin, and Yujia is on the upper main channel of the Puli River basin. Drainage area of Wenquan station is 1098 km^2^, and 366 km^2^ for Yujia station (Table 1).

**Table 1:** Basic geo-information of Dong River and Puli River basins.

	Area* (km^2^)	Minimum (m)	Maximum (m)	Average (m)	Std**(m)	Slope (degree)
Dong River	1098	192	2569	1155	560	23.2
Puli River	366	267	1326	627	213	12.1

Note: *Area means the basin area of runoff observation stations,

**Std means the standard error of the elevation.

Digital elevation model data at resolution 90 m (Shuttle Radar Topography Mission) were used to generate topographic information for the SWAT model. A land-use map extracted from Landsat Thematic Mapper data [21] and a soil map were used to generate hydrologic response units (HRUs), which were used as basic cells to calculate parameters in SWAT. Land-use types were reclassified into nine types [21]. Basin soil data were identified from the Chinese national 1∶1,000,000 scale soil map including five soil types [21], [22].

Daily climate inputs for SWAT included precipitation, minimum and maximum air temperature, and wind speed. The air temperature and wind speed data were collected from meteorological stations within the study area (Figure 1). Details of the various precipitation data are introduced in Section 2.2. Additional climate variables such as solar radiation and relative humidity were produced from a weather generator, using values from the nearest standard weather station [21], [22].

### Various Precipitation datasets

#### Monitoring Precipitation data

The monitoring precipitation data were acquired from a local observed agency. These were from four stations in the Dong River basin and three in the Puli River basin (Table 2). The period for all stations is 1 January 2000 to 31 December 2006.

**Table 2:** Basic geo-information of precipitation observation stations in the Dong River and the Puli River basins.

Dong River	Abbreviation	Latitude	Longitude	Elevation(m)
Da Jin	DJ	31.5178	108.4533	912
Guan Mian	GM	31.5365	108.694	1204
Wen Quan	WQ	31.3145	108.4744	225
Yan Shui	YSH	31.4313	108.694	1176

Puli River		Latitude	Longitude	Elevation(m)
He Xing	HX	30.7701	107.9229	480
Qiao Ting	QT	30.8355	108.1262	574
Yu Jia	YJ	30.8799	108.0234	520

#### NCEP-CFSR data

The NCEP-CFSR data is available for 1979–2013. They were designed and implemented as a global, high-resolution coupled atmosphere-ocean-land surface-sea ice system to provide the best estimate of the state of these coupled domains in the period [1]. The optimum interpolation algorithm of Xie et al. [23] is employed to partially account for the orographic enhancements in precipitation [1]. Although the original NCEP-CFSR data were based on a four-times-per-day step, the official website of the SWAT model (http://globalweather.tamu.edu/) supplied daily assimilation data for global application, as with the APHRODITE data, and the period of study was also 1 January 2000 to 31 December 2006. Spatial resolution of the NCEP-CFSR data is 0.5 degrees. See File S1 for the detail NCEP-CFSR data of the test basins.

#### APHRODITE data

The APHRODITE project created continuous daily gridded precipitation data for 1951–2007. These data cover monsoon Asia, the Middle East, and Russia. Version V1101R1 data of monsoon Asia were used in this study, with spatial resolution of 0.25 degrees [3]. The available data period was 1 January 1979 to 31 December 2007. The data cover the entire Pengxi River basin and the period 1 January 2000 to 31 December 2007 (Figure 1). The APHRODITE data combined the monitoring data in mountain areas and the Parameter-Elevation Regressions on Independent Slopes Model (PRISM) monthly precipitation climatology to correct the bias caused by orographic effects of Xie et al. [23]. File S2 list the APHRODITE data of the test basins in this study.

#### Trend surface data

Data of the land surface model forcing field for mainland China during 1958–2010, was provided by Li et al. [5]. We used the precipitation data from this dataset, with temporal and spatial resolutions daily and 0.05 degrees, respectively. The period is the same as that of the other data. The trend surface dataset applied the thin smoothing spline method to construct the surface trend of the precipitation; however, limited to the available of observed precipitation data, the trend surface product of precipitation didn't include the correction of elevation [5]. File S3 list the trend surface data of the test basins in this study.

## Methodology

### Soil Water Assessment Tool

SWAT is a temporally continuous, semi-distributed hydrologic model that was used to represent hydrologic and water quality processes in various basins with different temporal and spatial scale [24]. SWAT subdivides a watershed into subbasins connected by a stream network, and further delineates HRUs with unique combinations of land cover and soils in each subbasin. The hydrologic routines within SWAT account for snowfall and melt, vadose zone processes (infiltration, evaporation, plant uptake, lateral flows, and percolation), and groundwater flows [25], [26]. A modified SCS curve number method is employed to simulate the river runoff in the SWAT model, the Evapotranspiration (ET) is mainly calculated by using the Penman-Monteith method [25]. Moreover, the river channel routing of SWAT model is calculated by using a variable storage method [26].

### Model calibration settings

We used a daily simulation step for the SWAT model. The observed data is from 2002 to 2006 in Dong River basin, and it is from 2002 to 2004 in Puli River basin. Given a lack of river discharge observed data in the study area during the 2000s, we only conduct the validation in Dong River basin from 2005 to 2006. For the Dong River basin and Puli River basin, the calibration period is from 2002 to 2004.

We used the SWAT model calibration software SWAT-CUP 4.3.7 [27] to perform auto-calibration in the two study areas. SWAT-CUP is a public domain program, and thus can be copied and used freely. Among the model calibration methods available with SWAT-CUP, SUFI-2 has proven effective for calibrating and validating the SWAT model in different regions, with limited computational cost and high accuracy [27], [28]. In our study areas, applicability of the SUFI-2 method was also demonstrated by Wang et al. [21] and Yang et al. [22] for runoff and water quality modeling.

Results of hydrologic models are mainly controlled by two factors, input data and model structure, which is usually indicated by its parameterization. Thus, to conduct a “pure” evaluation for various precipitation data, we followed these rules:

1. The initial sampling ranges of the parameters were the same for both basins (Table 3)
2. Parameter sets generated by the Latin hypercube method [27] were the same for all precipitation data in each test basin
3. Repetition of SWAT-CUP was fixed at 1000 times for different input rainfall data

**Table 3:** Parameters used for auto-calibration of the two test basins.

Parameters	Type	Min	Max	Meaning
ALPHA_BF	v*	0	1	Base-flow alpha factor (days)
ALPHA_BNK	v	0	1	Base-flow alpha factor for bank storage
CH_K2	v	5	130	Effective hydraulic conductivity (mm/h)
CH_N2	v	0	3	Manning's n value for main channel
CN2	r**	−0.3	0.3	Initial SCS CN II value
ESCO	v	0.5	1	Soil evaporation compensation factor
GW_DELAY	v	0	30	Groundwater delay (days)
GW_REVAP	v	0	0.2	Groundwater “revap” coefficient
OV_N.hru	v	0	0.8	Manning's “n” value for overland flow
SOL_AWC	r	−0.3	0.3	Average available water
SOL_BD	r	−0.3	0.3	Soil Bulk Density
SOL_K	r	−0.3	0.3	Saturated conductivity
SURLAG	v	4	7	Surface runoff lag time (days)

Note: * v means actual value of calibrated parameter, and ** r means its relative range.

### Evaluation of Model Performance

#### Parameter sensitivity

We used 18 parameters for model calibration (Table 3). The parameter sensitivity analysis method of SWAT-CUP is based on the Latin hypercube [29], [30] and multiple regression methods. The multiple regression equation is as followed:
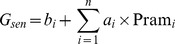
(1)


Here, 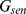 is the value of evaluation index for the model simulations, 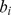 is a constant in multiple linear regression equation 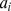 is a coefficient of the regression equation, 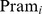 is a parameter generated by the Latin hypercube method, and n is the amount of parameters. The Latin hypercube method ensures that the full range of all parameters has been sampled. The multiple regression method that assigns changes of each model run output can be unambiguously attributed to the changed parameters [27], [31]. The *t-test* of this equation is applied to indicate the sensitivity of each parameter 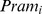.

#### Evaluation of runoff simulation

The Nash Sutcliffe Efficiency (NSE) [32] and coefficient of determination (R^2^) were chosen as indexes to evaluate the performance of various precipitation data for driving the hydrologic model. NSE and R^2^are calculated as
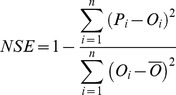
(2)

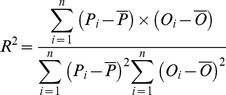
(3)


Here, 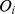 is observed runoff on day *i*, 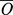
is average observed runoff, 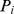
is the simulated value on day *i*, and 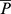
is average simulated runoff. The *NSE* is also used as the evaluation index (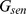) for the parameter sensitivity analysis in the Eq.(1).

#### Evaluation of model uncertainty

In SWAT-CUP, output uncertainty is quantified by the 95% prediction uncertainty band (95PPU), calculated at the 2.5 and 97.5% levels of the cumulative distribution function of the output variables [27], [28]. Two indices, the *r-factor* and *p-factor*, are used for evaluating output uncertainty based on the 95PPU. The *r-factor* is calculated by
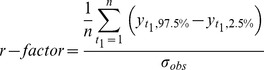
(4)


Here, 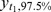 and 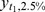 represent the upper and lower boundaries of the 95PPU, 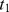 is the observed data and 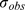 symbolizes the standard deviation of the measured data [28]. The *p-factor* is the percentage of observed data bracketed by the 95PPU band, and the *r-factor* stands for thickness of the uncertainty band [28].

The threshold for distinguishing the behavioral and non-behavioral simulation was set to 0.5 for NSE. Optimal calibration and parameter uncertainty for both methods was measured on the basis of proximity of the *p-factor* to 100% and *r-factor* to 1 [28].

## Results

Based on the input data, results of watershed delineation and weather station distribution are listed in Table 4. There are 37 subbasins of the Dong River basin and 13 subbasins in the Puli River basin. Based on nearest distance, which is calculated using station location and geographic centers of the subbasins, the SWAT model automatically assigns precipitation stations to each subbasin. The amounts of precipitation data in the two test basins are shown in Figure 1.

**Table 4:** Station numbers in the two test basins.

Test basin	Monitoring	NCEP	APHRODITE	T-S
Dong River	4	4	5	32
Puli River	3	2	3	12

Note: *NCEP stands for NCEP-CFSR data and **T-S for trend surface data.

### Station validation and spatial distribution of various precipitation data

#### Station validation

We also used four stations in the Dong River basin to evaluate accuracy of the various precipitation data. Three different evaluation indices were determined: 1) correlation efficiency (R), 2) mean error (ME), and 3) mean absolute error (MAE). Equations for these indices are
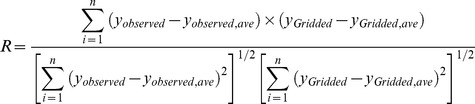
(5)

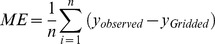
(6)

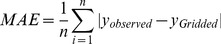
(7)


Here, 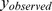 is observed precipitation data on day *i*, 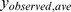 is the average value of observed precipitation data, 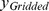 represents the gridded precipitation data on day *i*, 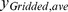 is the average value of the gridded precipitation data, and *n* is the total number of observed values.

The observation station and nearest station were used for comparison. The results are presented in Table 5. The results of R for the Dong River basin show that at all four stations, NCEP-CFSR had a satisfactory performance compared with the other two datasets. The APHRODITE and trend surface data achieved reasonable R values compared with the NCEP-CFSR data in Puli River basin. Regarding ME, the APHRODITE and trend surface data produced at all stations, a negative difference compared with the monitoring precipitation data indicated that the first two datasets underestimated the precipitation data at those sites. Regarding the NCEP-CFSR data, positive ME values at DJ and GM stations indicate overestimation of the precipitation. In contrast, at WQ and YSH stations, NCEP-CFSR underestimated the precipitation. The NCEP-CFSR data had the largest MAE values at all validation stations compared with the APHRODITE and trend surface data. For the latter two datasets, MAE for all stations was 2.50–4.0 mm, which was a small range compared with that of the NCEP-CFSR data.

**Table 5:** Evaluation results of different gridded precipitation datasets in the Dong River basin.

Station	DJ			GM			WQ			YSH		
	APHRODITE	NCEP*	T-S**	APHRODITE	NCEP	T-S	APHRODITE	NCEP	T-S	APHRODITE	NCEP	T-S
R	0.69	0.56	0.60	0.67	0.56	0.59	0.71	0.53	0.64	0.64	0.55	0.58
ME(mm)	−0.93	3.45	−0.69	−1.61	2.75	−1.42	−0.53	−0.88	−0.22	−1.76	−1.93	−1.43
MAE(mm)	3.18	6.24	3.64	3.71	6.32	4.10	2.14	2.55	2.49	3.72	4.10	4.03
F-test	2229.0	1042.6	1467.3	1688.9	1007.3	1166.7	1353.9	590.2	1019.7	1373.3	883.0	1132.8

There were three validation stations in the Puli River basin (Table 3). Results are presented in Table 6. The results of R for that basin show that APHRODITE data gave the best performance compared with the other two datasets. ME values of APHRODITE and trend surface data were positive, which means that the precipitation data were overestimated at the three stations. For the NCEP-CFSR data, ME values were negative at QT and YJ stations and positive at HX. MAE had the same tendency as the results for the Dong River basin. The APHRODITE and trend surface data delivered more accurate results relative to the NCEP-CFSR data.

**Table 6:** Evaluation results of different gridded precipitation datasets in the Puli River basin.

Station	HX			QT			YJ		
	APHRODITE	NCEP	T-S	APHRODITE	NCEP	T-S	APHRODITE	NCEP	T-S
R	0.66	0.40	0.61	0.64	0.43	0.59	0.67	0.41	0.62
ME (mm)	0.45	0.66	0.48	0.23	−0.38	0.38	0.03	−0.58	0.18
MAE(mm)	2.38	3.34	2.65	2.63	3.04	2.98	2.56	3.19	2.94
F-test	795.3	249.0	532.2	616.1	327.5	469.3	907.7	240.5	630.5

Note: *NCEP stands for NCEP-CFSR data and **T-S for trend surface data, the correlation analysis is tested in the significant level α = 0.05. For both basins, because the regression analysis number of data are all over 1000, thus, the F-test value at significant level α = 0.05 is adapted 3.84 for all linear regression analysis.

#### Spatial distribution of various precipitation data

**Figure 2:**
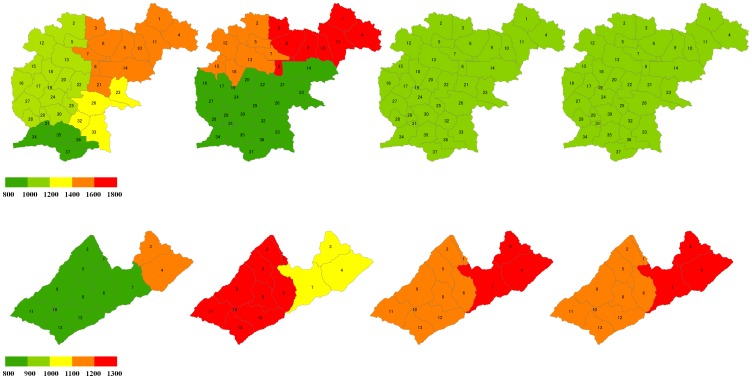
Spatial distribution of annual precipitation value for various precipitation datasets for the Dong River (up) and the Puli River (down) basins, from left to right is Monitoring data, NCEP CFSR data, APHRODITE and trend surface data.

**Figure 3:**
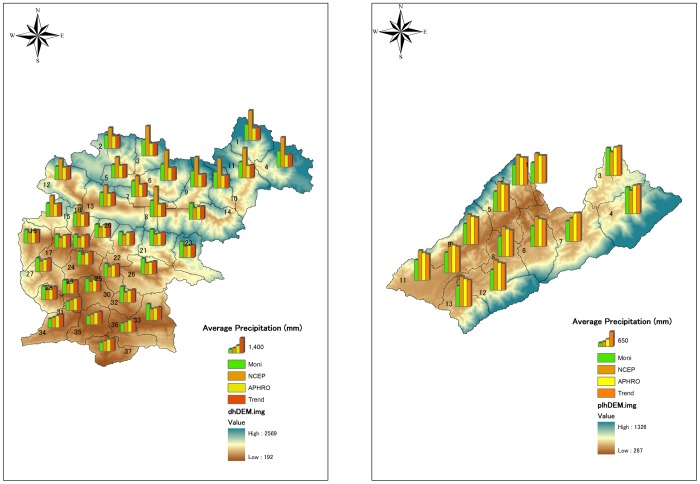
Annual precipitation value of various precipitation datasets for the Dong River (left) and the Puli River (right) basins.

The areal distributions of different precipitation datasets are shown in Figure 2 and 3. The annual precipitation distribution of in Dong River basin (Figure 2, left) indicate that the results of monitoring data and the NCEP-CFSR data present more variation compared with the APRHODITE data and the trend surface data. The precipitation value of Dong River basin calculated by the monitoring data is varied from 800 mm to 1600 mm. The value of NCEP-CFSR data indicates a more large variation, which is from 800 mm to 1800 mm. However, the results of APHRODITE data and the trend surface data show that the annual precipitation in Dong River basin is from 1000 mm to 1200 mm. In the Puli River basin, the monitoring precipitation data indicate that the annual precipitation amount is from 800 mm to 1200 mm in Puli River basin (Figure 2, right). As same as in the Dong River basin, the APHRODITE data and the trend surface data also perform similar results in Puli River basin. The range of annual precipitation of these two datasets is from 1100 mm to 1300 mm. Though the NCEP-CFSR also shows the annual precipitation is from 1100 mm to 1300 mm, the spatial distribution clearly indicate that it present contrary results compared with other three datasets. In the downstream of Puli River basin, the annual precipitation calculated from the NCEP-CFSR data is lower than other three datasets. However, in the upstream, the annual precipitation of NCEP-CFSR data present relative higher values compared with other datasets.

The annual precipitation value in the Dong River basin (Figure 3, left) shows that in the north and northwest portions, which have high elevations, the NCEP-CFSR data had positive differences compared with other data. This means that the input precipitation values for the SWAT model subbasin were much higher than other data. In contrast, in the downstream area of the Dong River basin, the NCEP-CFSR data had negative differences relative to the other datasets. For the APHRODITE and trend surface data, their differences with the monitoring data were greater in the upstream (high elevation) area, but smaller in the downstream area. In the Puli River basin (Figure 3, right), the monitoring and NCEP-CFSR data had sensible differences with the APHRODITE and trend surface data. The NCEP-CFSR data had positive differences compared with the APRHODITE and trend surface data; however, the monitoring data had negative differences.

Spatial distributions of the various datasets indicate that the input precipitation data varied greatly across the test basin. Such differences may lead directly to variations of simulation results and produce uncertainties in the SWAT model. However, because of the lack of observed precipitation data in each subbasin, we cannot directly evaluate the quality of the various datasets there. This situation is also inadequate for evaluating performances of the precipitation datasets based only on in situ validation and spatial distributions. Therefore, model calibration must be done.

### Results of daily runoff simulation

Results of parameter sensitivity analysis are shown in Table 7. It is clear that in both the Dong and Puli River basins, the most sensitive parameters were ALPHA_BNK, CH_K2, CH_N2, and CN2. However, from the average rank of each basin, the order of sensitive parameters is different. This order is ALPHA_BNK, CH_K2, CH_N2 and CN2 in the Dong River Basin. For the Puli River Basin, the average rank value clearly shows that CN2 was more sensitive than the other three parameters. There were no differences in sensitive parameters for the various precipitation datasets, and the other parameters were not sensitive to the hydrologic simulation. In the Dong River basin, the monitoring precipitation data and the gridded data obtain very similar results, except the 4^th^ rank of sensitive parameter. However, the sensitive rank in the Puli river basin show that the gridded datasets presents more consistent results compared with the monitoring data, especially for the CH_K2 and CH_N2, the ranks are different in monitoring data and gridded data. Moreover, compared with the two test basins, the gridded precipitation data can obtain similar sensitive parameter ranks rather than the monitoring data.

**Table 7:** Ranking of sensitivity of various parameters for the Dong River and the Puli River basins, based on auto-calibration of SWAT-CUP.

		Dong River					Puli River			
Parameter Name	Monitoring	APHRODITE	NCEP*	T-S**	Average	Monitoring	APHRODITE	NCEP	T-S	Average
ALPHA_BF	8	8	11	9	9	11	6	5	5	6.75
**ALPHA_BNK**	1	3	2	2	**2**	1	2	2	2	**1.75**
**CH_K2**	3	2	1	3	**2.25**	6	3	4	3	**4**
**CH_N2**	2	1	3	1	**1.75**	5	4	3	4	**4**
**CN2**	5	4	4	4	**4.25**	2	1	1	1	**1.25**
ESCO	11	7	13	6	9.25	3	7	6	7	5.75
GW_DELAY	7	9	6	8	7.5	13	5	8	6	8
GW_REVAP	12	10	10	10	10.5	9	8	7	8	8
OV_N	9	12	8	11	10	8	10	11	12	10.25
SOL_AWC	13	13	12	13	12.75	10	13	9	13	11.25
SOL_BD	6	6	7	7	6.5	7	12	13	9	10.25
SOL_K	4	5	5	5	4.75	4	9	10	10	8.25
SURLAG	10	11	9	12	10.5	12	11	12	11	11.5

Note: *NCEP stands for NCEP-CFSR data and **T-S for trend surface data.

**Figure 4:**
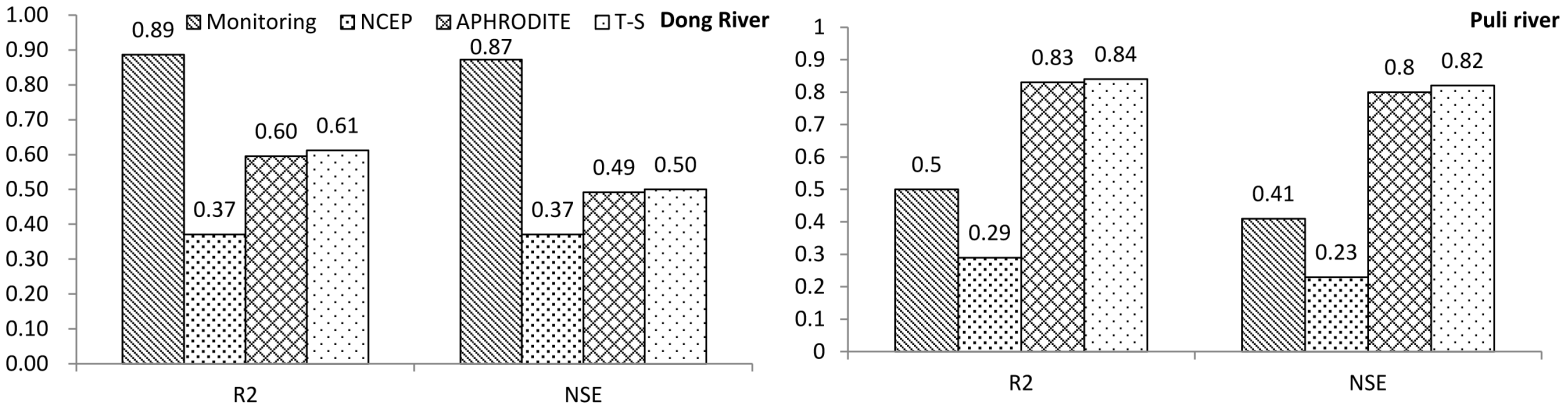
R^2^ and NSE of various precipitation datasets for the Dong River and the Puli River basins during the calibration period.

**Figure 5:**
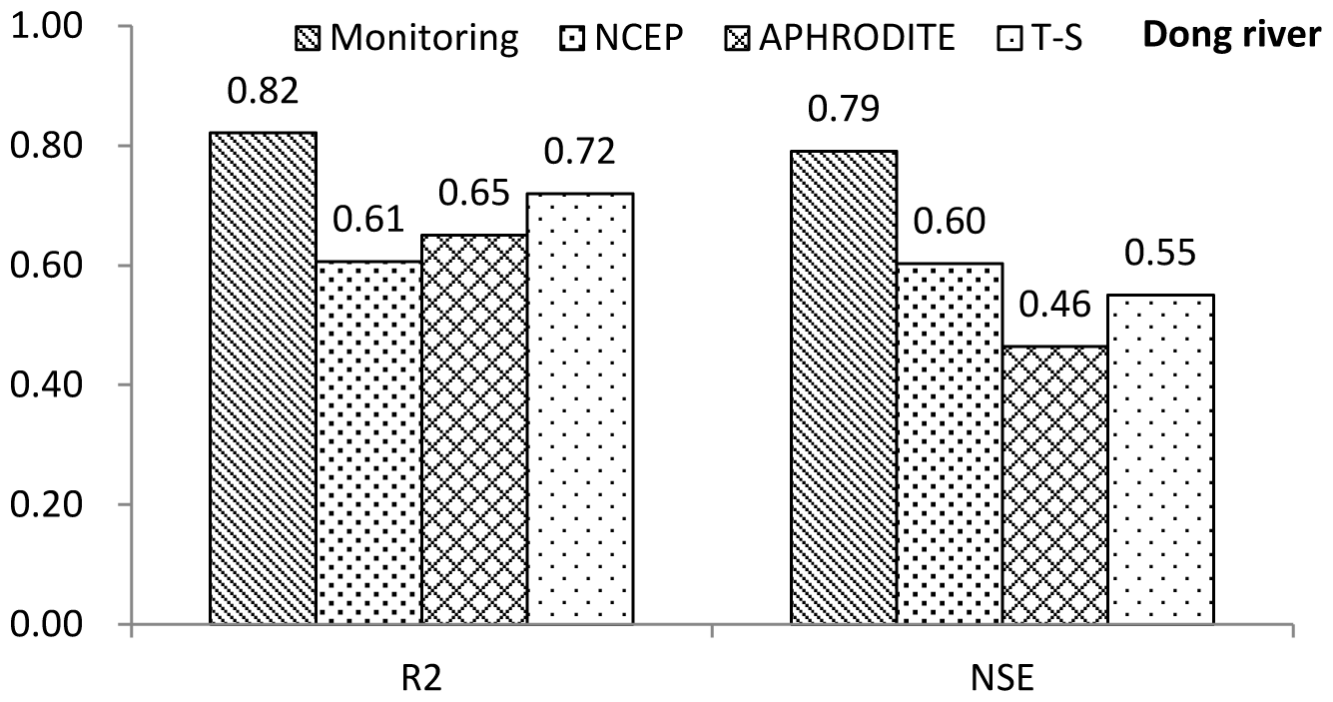
R^2^ and NSE of various precipitation datasets for the Dong River during the validation period.

**Figure 6:**
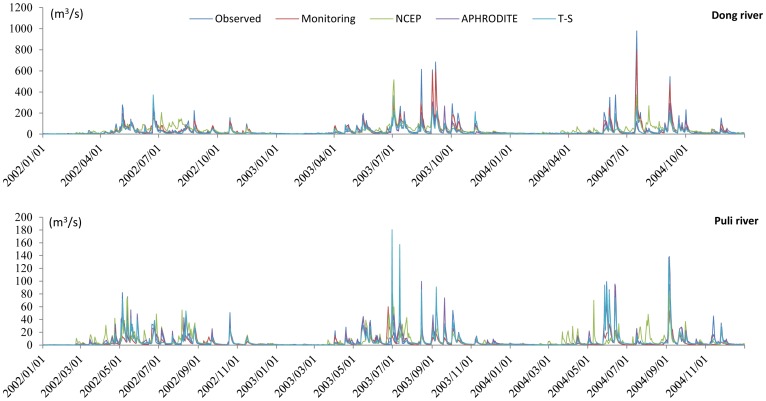
Simulation hydrographs of various precipitation datasets for the Dong River (up) and the Puli River (down) basins during the calibration period. NCEP stands for NCEP-CFSR data and T-S for trend surface data.

**Figure 7:**
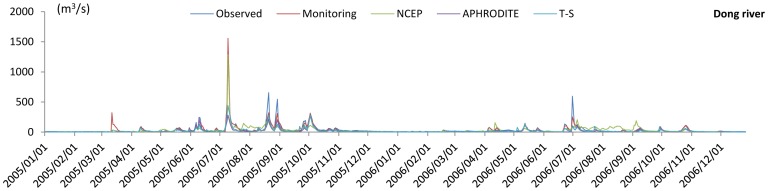
Simulation hydrograph of various precipitation datasets for the Dong River basin during the validation period. NCEP stands for NCEP-CFSR data and T-S for trend surface data.

Daily runoff simulation results for the Dong River basin and Puli River basin are shown in Figures 4 and 5, respectively. For the Dong River Basin, during the calibration period, R^2^ is 0.89 and NSE is 0.87 of the monitoring precipitation data. For NCEP-CFSR data, R^2^ is 0.37 and NSE is 0.37, which show a significant decrease relative to the monitoring data, and are lower than the threshold of behavioral simulation (NSE = 0.5). R^2^ and NSE are 0.60 and 0.49 for APHRODITE data, respectively. Although these values are higher than for the NCEP-CFSR data, they are under the threshold for behavioral simulation assessment. The trend-surface data show similar results to the APHRODITE data, with a slight decrease. NSE of the trend surface data exceeded the behavioral threshold value (NSE = 0.50; R^2^ = 0.61). During the validation period, the R^2^ and NSE values of monitoring data presents relatively good results (R^2^ = 0.82; NSE = 0.79), for the NCEP-CFSR data, the performances are improved (R^2^ = 0.61; NSE = 0.60). In the results of APHRODITE data, the R^2^ is 0.65 which is slightly improved compared with the calibration period; however, the NSE slightly decreases to 0.46. For the trend surface data, the performances (R^2^ = 0.72; NSE = 0.53) of trend surface data during the validation period are also improved compared with the calibration period. The hydrograph for the Dong River basin (Figure 6, up, and Figure 7) demonstrated that only the monitoring precipitation data could reproduce the hydrograph well during flood periods, compared with the various gridded datasets.

For the Puli River basin (Figure 4), R^2^ is 0.83 and NSE is 0.80 for APHRODITE data, and are respectively 0.84 and 0.82 for the trend surface data. For the monitoring data, the indices are R^2^ = 0.50 and NSE = 0.41. R^2^ and NSE were 0.29 and 0.23, respectively for the NCEP-CFSR data, both of which represent significant decrements relative to the other two datasets. The hydrograph (Figure 6, down) shows that the peak flow and sequence in the Puli River basin matched well for the APHRODITE and trend surface data, compared with the monitoring data and NCEP-CFSR data.

### Model uncertainties induced by various precipitation data

**Figure 8:**
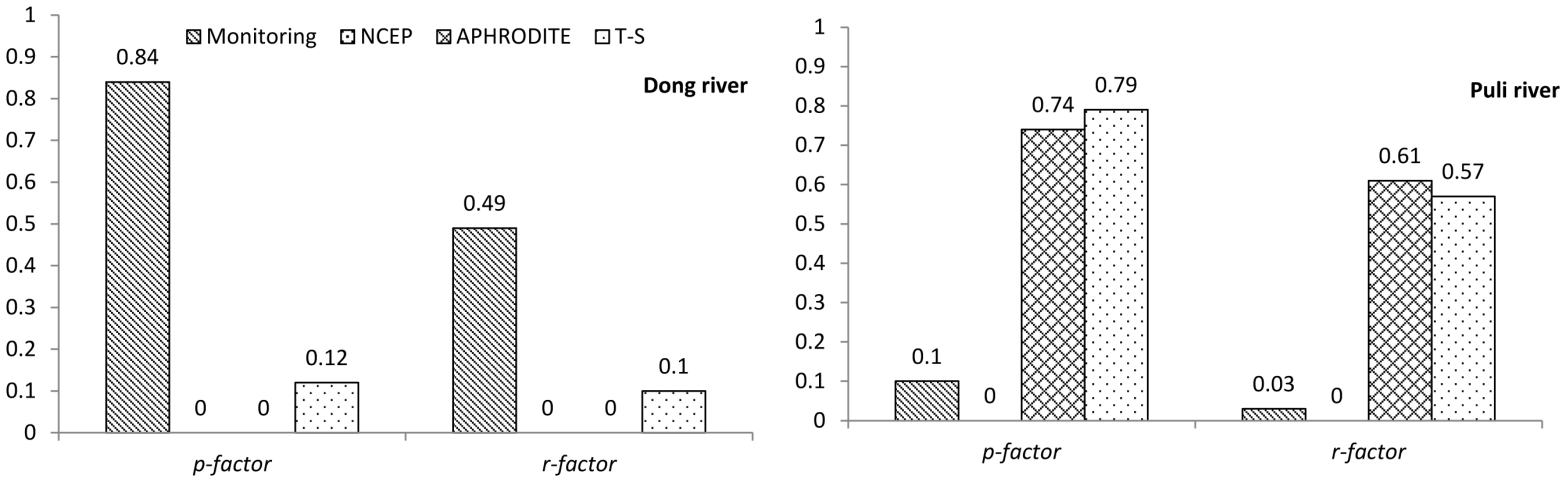
*p-factor* and *r-factor* of different precipitation datasets for the Dong River and the Puli River basins. NCEP stands for NCEP-CFSR data and T-S for trend surface data.

The *p-factor* and *r-factor* results are shown in Figure 8. Trends of the factors are the same as R^2^ and NSE. In the Dong River basin, the monitoring precipitation data gave the best results. Because there was no simulation result in excess of the behavioral threshold, *p-factor* and *r-factor* values were set to zero for both NCEP-CFSR and APHRODITE data. The results of both factors for the trend surface data show that they had greater uncertainties compared with the observed data.

In the Puli River basin (Figure 8), the APHRODITE and trend surface data yielded more preferable results relative to the monitoring and NCEP-CFSR data. Because there was no simulation result in excess of the behavioral threshold, *p-factor* and *r-factor* values were set to zero for the NCEP-CFSR data. For the monitoring data, although they obtained behavioral simulations, the *p-factor* and *r-factor* values still demonstrate that they led to more uncertainties relative to the APHRODITE and trend surface data.

## Discussion

### Performances of various precipitation data upstream of TGR

In situ validation performances of the various precipitation datasets for the Dong and Puli basin show that APHRODITE and trend surface data can reflect the basic quantity of precipitation, as determined by R, ME, and MAE. The NCEP-CFSR data always gave larger errors compared with the other two datasets. Additionally, the APHRODITE data were more accurate than the trend surface data; however, the differences were not significant. As presented, the monitoring data were accurate but could only represent precipitation in or near the station. Consequently, to evaluate the performance of the different datasets, we should also rely on the model results.

The daily runoff simulation results of the SWAT model proved that the APHRODITE and trend surface data could reproduce the hydrologic processes in both the Dong and Puli basin more effectively than NCEP-CFSR. The model uncertainty results also suggest that the latter dataset has greater uncertainties compared with the other two. Amongst all the gridded datasets, the trend surface data always achieved the best results, as determined by both monitoring data and hydrologic model testing. The spatial distribution results already demonstrated that the NCEP-CFSR data gave significant differences compared with the other two datasets and the monitoring data. Section 4.2 revealed that the significant spatial distribution differences of the datasets can lead to variable simulation results. Further, the model uncertainty results indicate that the spatial distribution of NCEP-CFSR data may directly cause a relatively weak performance and greater uncertainty in the SWAT model.

Compared with sparse and isolated observation stations, the gridded data have an advantage in considering effects of station surroundings for prediction at a fixed location [1], [24]. Thus, the consideration of impacts from surrounding stations in APHRODITE and trend surface data products may make them more valid and permit more robust data input to SWAT model runoff determination at TGR, compared with the NCEP-CFSR data. The data source for the latter dataset is global public observation stations [1]. Thus, compared with the APHRODITE dataset that covers Asia with over 40,000 stations [3], [24], the NCEP-CFSR data are too sparse to achieve accurate reanalysis results in our study area. Li et al. [5] explained that the trend surface data are also based on public global exchange stations, but the present simulation results suggest that the trend surface interpolation method and its products may be more applicable to our study area.

Nevertheless, we recognize that the performances of the various gridded precipitation data were weak relative to the monitoring data for the Dong River basin, where there are large elevation differences. As mentioned above, in micro-scale or meso-scale basins, precipitation can be influenced by various factors such as topography, wind speed, and hill aspects. Given this, the gridded precipitation data may lose the capacity to reflect the precipitation distribution accurately. Thus, we also addressed the applicability of various precipitation datasets by considering topographic influences and data density.

### Applicability of different precipitation data: topography

If the topography strongly affects precipitation, strong linear relationships can be obtained by analyzing elevation and precipitation differences among stations [33], [34]. The “precipitation lapse rate” is used to evaluate this relationship. We used this rate to assess the precipitation influence in each basin, using
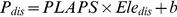
(8)


**Figure 9:**
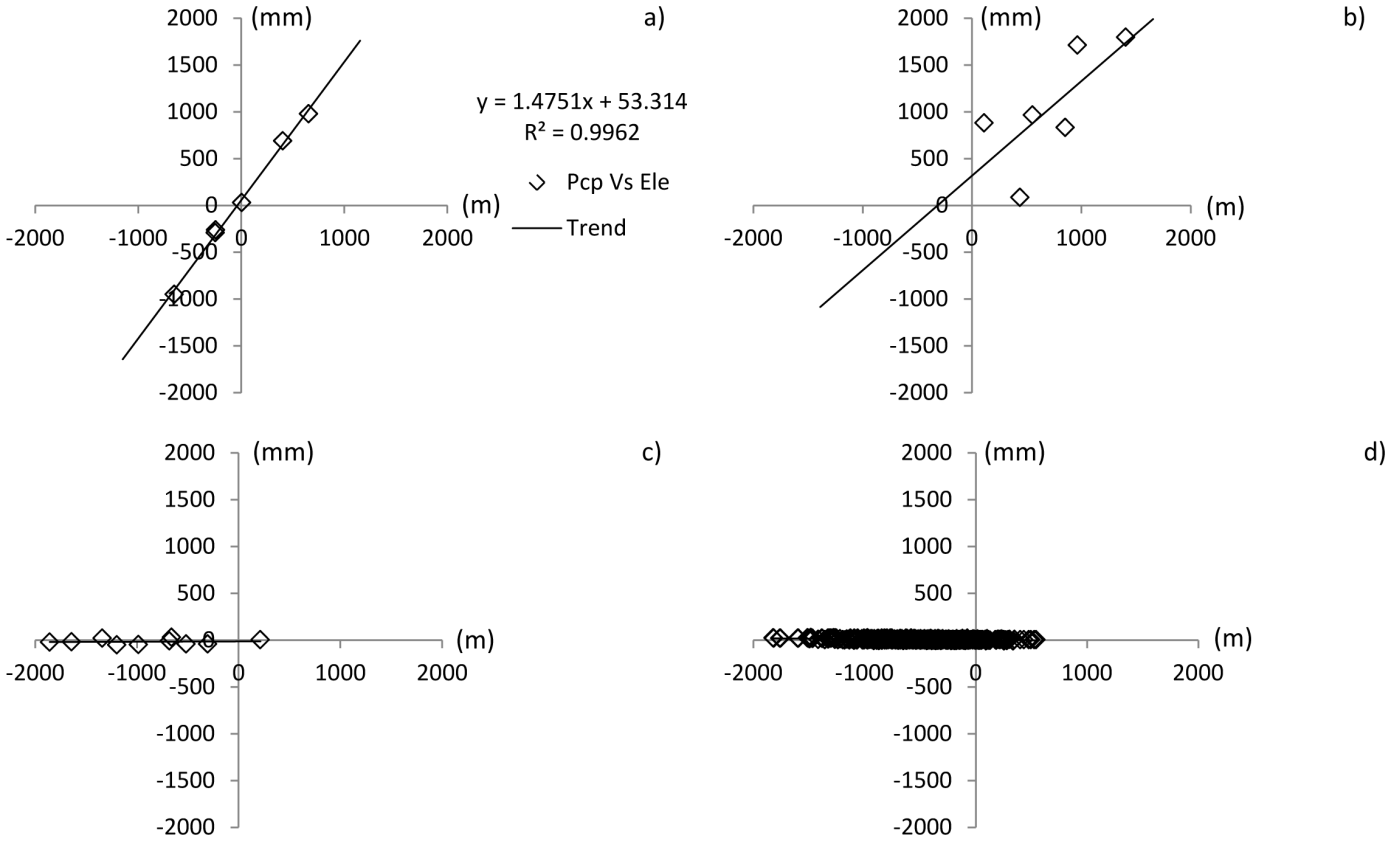
Correlation between elevation difference and precipitation difference in the Dong River basin (significant level α = 0.05). a) Monitoring data; b) NCEP-CFSR data; c) APHRODITE data; d) trend surface data.

**Figure 10:**
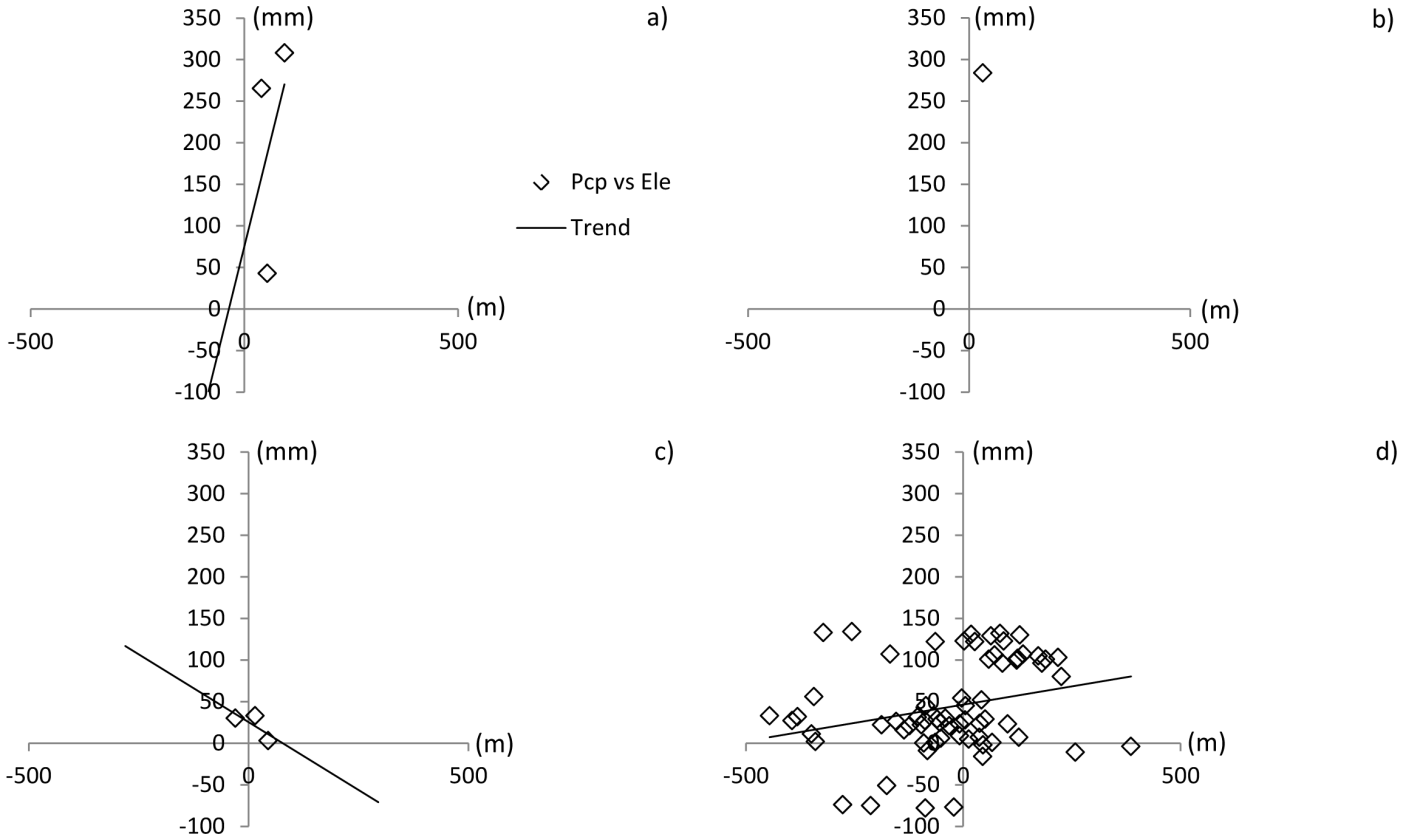
Correlation between elevation difference and precipitation difference in the Puli River basin (significant level α = 0.05). a) Monitorin data; b) NCEP-CFSR data (only one point available, so linear regression analysis is missing); c) APHRODITE data; d) trend surface data.

Here, 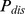 is the precipitation difference between a pair of stations (the station pair is determined by permutations of all available stations used in the SWAT model), 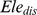 is the elevation difference between the pair, 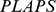 is the first order or slope of the linear regression equation, and 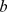 is the intercept of the linear equation. For both Dong River basin and Puli River basin, the linear regression analysis is conducted with the F-test in significant level α = 0.05 (Table 8). The results are presented in Figures 9 and 10.

**Table 8:** F-test of linear regression for elevation difference and preciptiaiton difference of all datasets in Dong River basin and Puli River basin.

	Dong River basin				Puli River basin			
	Monitoring	Aphrodite	NCEP	T-S	Monitoring	Aphrodite	NCEP	T-S
N*	6	10	6	496	3	3	Null	66
F_0.05_**	7.71	5.32	7.71	3.84	7.71	7.71	Null	3.84
F_re_***	1047.8	0.05	4.31	242.49	0.2	Null	Null	4.57

Note: *N stands for the number of data used for linear regression, **F_0.05_ stands for the F-test value at significant level α = 0.05, F_re_ stands for the F-test value calculated from the regression analysis. For the NCEP-CFSR data in Puli River basin, because only one point is available, thus the linear regression is missing.

In the Dong River basin, R^2^ of the linear regression equation for the monitoring data (Table 8, Figure 9a) was nearly 1, clearly indicating a strong correlation between elevation and precipitation differences. Conversely, judging by the F-test, there is no correlated relationship between elevation and precipitation for APHRDOITE and NCEP-CFSR data. Though the F-test value is larger than its value in the significant level (Table 8), the R^2^ of trend surface data indicate the linear regression relationships between elevation and precipitation is weak results in Dong River basin. (Figure 9 b), c) and d)). In the Puli River basin, the linear regression result at the significance level 0.05 ((Table 8, Figure 10) shows there is no significant linear relationship between elevation and precipitation data for all the precipitation datasets in this basin.

Moreover, basic geo-information of the two test basins (Table 1) confirms their different elevations. In the Dong River basin, the minimum elevation is 192 m and maximum 2569 m; its standard error is 560 m and the slope is 23.2 degrees. In the Puli River basin, the minimum elevation is 267 m and maximum 1326 m, with standard error 213 m and slope 12.1 degrees. Obviously, the topography has greater variation in the Dong River basin than in the Puli River basin.

Considering the simulation results of Section 4.2 in the Dong River basin, the results of simulation and uncertainty clearly demonstrate that the monitoring data gave results superior to those of the gridded data. Significant differences of precipitation lapse rate of monitoring precipitation data strongly suggest that for Dong River basin, topography is the main influence on the spatial distribution of precipitation data, which thereby affects SWAT model simulation results. However, in the Puli River basin, all data indicate that topography is not the main influence on precipitation variation. Uhlenbrook et al. [35] indicated that the topographic influence on the rainfall will also significantly influence the results of hydrological model. Weisse and Bois [36] also demonstrated that topographic characterization has significant influence on the rainfall, especially the heavy rainfall in the French Alps. Sanchez-Moreno et al. [37] also presented the topography can significantly influence the trend of monthly and seasonal precipitation distribution in Santiago Island, Cape Verde. In our study, the results of Dong River basin also clearly demonstrated that the gridded precipitation data cannot reflect the rainfall distribution which is greatly influenced by the topography. Contrarily, in Puli River basin, of which the precipitation receives less influence form the topographic, the simulation results indicate that the gridded data can reflect the value and distribution of precipitation more effectively compared with the sparse monitoring data.

As presented in the section 2.2, except the trend surface method, the NCEP-CFSR data and the APRHODITE data are all employed the optimum interpolation method for correction of Topographic. However, our results indicate that the effects of the correction datasets on local regions still cannot match the applicable of SWAT model. Moreover, Silva et al. [38] indicate that the NCEP CFSR data may overestimate the precipitation in the coastal mountain area in Brazil. Andermann et al. [39] shown that for APHRODITE data also it can present relatively good performances in yearly and monthly steps in mountain areas of Himalaya front, and its performances in daily step is still need to be improved. Ono et al. [40] also reported that for the heavy rainfall investigation in Mekong River, the local topography is still need to be considered as a correction factor for improving the accurate of APHRODITE data. The results of our research indicate that for their applications in local regions, the applicability, especially, topographic effects should be considered and discussed firstly.

### Applicability of various precipitation data: spatial density

In addition to topography, input precipitation data density is an influence, and alters the hydrologic model results. Basic information on station density is given in Figure 1 and Table 4. In Dong River Basin, the NCEP-CFSR and APHRODITE data have the same spatial density as the monitoring data. The trend surface data, from more than 30 stations for the simulation, gave a poor result compared with monitoring data. In the Puli River Basin, those data were from 12 stations and the APHRODITE data only three stations, but the performance of these data showed no significant difference. Additionally, station density was similar for monitoring (three stations) and NCEP-CFSR (two stations) data. Compared with the APHRODITE data, however, those two datasets had less capacity to portray the hydrologic processes in the Puli River Basin.

The gridded data usually contain precipitation data of high density for simulation, relative to large basins. However, the performances of the hydrologic simulations in this study strongly suggest the spatial density has no major influence on model simulations of this study area. Data accuracy and representativeness is more important. Additionally, these gave auxiliary proof that topography is the dominant influence in the study basin.

## Conclusions

The objective of the present study was to evaluate the capabilities of various gridded precipitation datasets (NCEP-CFSR, APHRODITE, and trend surface) for the area upstream of TGR, using both monitoring data and a hydrologic model.

Two test basins (Dong and Puli) were selected for data evaluation. Comparisons between the gridded and monitoring precipitation data suggest that APHRODITE data can explain the precipitation more accurately than the other two datasets at observation stations. For model testing in the Dong River basin where there is significant elevation variation, the simulation results suggest that all gridded precipitation data had reduced abilities to drive SWAT for achieving reasonable results. However, in the Puli River basin with relatively flat topography, the APHRODITE and trend surface data can give more accurate results than the NCEP-CFSR data, and even better than the monitoring data.

The results also prove that the APHRODITE and trend surface data are more stable and applicable than the NCEP-CFSR data in the study area. The results also suggest that in the application of the hydrologic or other models to the TGR using the gridded precipitation datasets, one must consider topographic effects on those data.

## Supporting Information

File S1 **Extracted precipitation data of NCEP-CFSR data in the test basins for SWAT model.**
(ZIP)

File S2 **Extracted precipitation data from APHRODITE data in the test basins for SWAT model.**
(ZIP)

File S3 **Extracted precipitation data from trend surface data in the test basins for SWAT model.**
(ZIP)
